# Bridging Personal and Population in Excitability Diseases: Will Studies of Rare Diseases Bring Generalizable Mechanisms From Monogenic Channelopathies?

**DOI:** 10.1093/function/zqab072

**Published:** 2022-01-04

**Authors:** Colin G Nichols, Conor McClenaghan

**Affiliations:** Center for the Investigation of Membrane Excitability Diseases, and Department of Cell Biology and Physiology, Washington University School of Medicine, St. Louis, MO 63110, USA; Center for the Investigation of Membrane Excitability Diseases, and Department of Cell Biology and Physiology, Washington University School of Medicine, St. Louis, MO 63110, USA

## The Gap: What is Missing Between Monogenic Disorders and the Big Diseases?

Twenty years ago, Sir David Weatherall wrote “As a result of … sequencing of … the human genome, the role of genetics in the pathogenesis of human disease now dominates biomedical research. There is every sign that the rapidly evolving technology of the post genome era will unravel the function of the human genome and explain how the 50,000 to 100,000 genes interact with one another and the environment to make us what we are”.^1^ Has it? Arguably, not. Weatherall went on to say “Further exploration of the genome may also provide information on some of the common killers of Western society, such as heart disease, stroke, diabetes, and psychiatric disease”, and then “more important, and certainly more complex, will be relating genotype to phenotype. Many of our most important diseases … reflect varying susceptibility, due to the action of many different genes and a wide variety of environmental factors and to the ill understood biology of ageing.” We have indeed witnessed a burgeoning of identified monogenic disorders, associated with some spectacular therapeutic improvements, but how much have these monogenic developments changed our thinking about how organs function, and how we view ‘common’ diseases, which are not obviously driven by specific genes, but might ‘run in families’?

Through consideration of the clinical diversity of β-thalassaemias that result from primary mutation of the β globin locus, Weatherall showed how variability at loci having nothing to do with hemoglobin production, as well as environmental factors, could cause extremely variable outcomes and turn what at first sight seems a simple monogenic disorder into an extremely complex syndrome.^1^ This expansionist phenotypic nature of single gene defects might seem to make predictions from parallel outcomes of distinct gene defects a futile exercise. However, we would suggest that it may be possible to integrate convergent findings from different monogenic disorders to develop paradigms that may explain polygenic diseases. This could be especially so for ion channel pathologies and, in keeping with the theme of asking what is ‘round the corner’ for physiology, we would argue that we may be on the cusp of significant advances in such understanding.

## Insights From Monogenic K Channel Diseases: Examples of Unpredicted Outcomes

Ion channels underlie action potentials, and so, of course, many ion channel gene mutations result in predictable excitability changes in relevant systems—the nervous system and heart being obvious places. Understanding the roles of specific channels in epilepsy syndromes^2^ and cardiac arrhythmias^3^ is and will continue to be increasingly sophisticated. At least 16 distinct congenital long QT syndromes are now recognized as a result of mutations in multiple voltage-gated K, Ca, and Na channel genes, but interestingly also as a result of mutations in non-channel genes, including those for Ankyrin (*ANK2*, LQT4), A-kinase anchor protein (*AKAP9*, LQT1), and alpha-1 syntrophin (*SNTA1*, LQT12).^3^ Such findings indicate a synergy with the ion channel substrate, and can spur further insights to molecular complexes and convergent signaling pathways.

Our own recent focus on the consequences of gain- (GOF) and loss-of-function (LOF) mutations in the cardiovascular K_ATP_ channel genes *ABCC9* and *KCNJ8* has illustrated consistent syndromic consequences of each in mice, and thereby informed the aligned human Cantu and AIMS syndromes, respectively.^4^ Significantly, many key features of Cantu Syndrome, including massively enlarged and hypercontractile hearts, as well as hypertrichosis, are not naively predicted from our classical understanding of K channel function in the cardiovascular system. It turns out that, rather than causing cardiac action potential shortening, and short QT syndrome, for instance, the mutational consequences are probably most directly relevant in vascular smooth muscle. Here, K_ATP_ GOF does indeed cause the predicted vasodilation and reduced systemic vascular resistance but this then leads to enhanced renin–angiotensin signaling, which drives cardiac enlargement.^5^

The mechanistic causes of hypertrichosis (excess hair growth) are still not clear, but the hair-growth promoting effects of minoxidil (the active ingredient in Rogaine©) can at least now be squarely placed on its action to activate K_ATP_. Individuals with Cantu syndrome also all have a characteristic facial ‘dysmorphology’, essentially a broad nasal bridge, wide mouth with full lips, and macroglossia. Again, the underlying cause is not obvious, but may also be related smooth muscle underactivity, perhaps in the lymphatic vessels that drain excess fluid from the peripheral tissues. Intriguingly, there is some convergence of outcomes of GOF mutations in other K channels.^6^ Bauer–Tartaglia syndrome and Zimmermann–Laband syndrome, linked to GOF mutations in the K2P TRAAK channel *KCNK4* gene, and the human EAG-like *KCNH1* or the small-conductance Ca-activated SK3 channel *KCNN3* genes, respectively, exhibit similar facial changes and generalized hypertrichosis. These parallels may hold important clues as to the underlying cellular mechanisms.

What about the prospect that the opposing actions of channels that promote excitability versus those that suppress excitability will result in parallel, but *a priori* unpredictable, outcomes for GOF in one and LOF in the other? LOF mutations in L-type Ca channel genes cause various neurological defects, including spinocerebellar ataxia (*CACNA1A)*, seizures (*CACNA1B*), or cardiac long QT syndromes (*CACNA1C*), while GOF in the latter causes Timothy syndrome, a very complex disorder in itself, with multiple organs affected.^7^ One interesting feature is syndactyly, which is also seen in Andersen–Tawil Syndrome ,^8^ caused by LOF mutations of the strong inward rectifier Kir2.1 channel gene (*KCNJ2*). Again, while neither outcome is immediately predicted from known roles of these channels, such parallels may be important clues to mechanisms.

## “Mind the Gap”: What of the Prospects for More Common Pathologies?

Mechanistic insights have been facilitated by the ease of generation of knockin/knockout animals—increasingly possible in multiple species—that has been made feasible in just the last few years by CRISPR/Cas9 and related genome manipulation technologies. Increasingly, in combination with older transgenic strategies, such approaches are readily available to ‘genomic physiologists' and going forwards we can expect accelerating insights for all genes. Many common diseases do not show a high level of heritability, and probably result from variable susceptibility to a wide range of environmental factors mediated by many different genes. While multiple ‘omic’ approaches can identify disease-related gene variants, transcripts, and proteins, the value of subsequent *in silico* pathway analysis for informing mechanisms is not so obvious. Instead, can the kinds of functional insights obtained from monogenic disorders say anything about common diseases? A cursory look at the monogenic consequences of changes in other ion channels expressed in vascular smooth muscle provides some intriguing findings. Pulmonary arterial hypertension (PAH) is a fatal and not uncommon cardiopulmonary disease associated with elevated pulmonary vascular resistance, and which is often resistant to current treatments. PAH has been associated with mutations in four ion channel genes (*KCNK3, ABCC8, KCNA5*, and *TRPC6*). LOF K channel mutations, but increased activity of *TRPC6*, points towards a convergent underlying electrical mechanism and, while ion channel involvement in other forms of PAH is not obvious, might reflect other genetic and environmental PAH triggers acting through ion channels.^9^

We still cannot predict the clinical course of even simple monogenic diseases, so the notion that we may be able to predict the occurrence of ‘common’ diseases based on genotype alone is probably fanciful. However, and belatedly recognized by many who have the misfortune to get clinically diagnosed with ‘common’ diseases, mechanistic understanding of them is still often very rudimentary. We would argue that we are in a golden era for physiologists, in which functional analysis of monogenic derangements can provide very powerful insights to monogenic disorders. Beyond that, novel mechanistic paradigms will at the very least broaden our understanding of physiology and may prove useful in improved treatments for ‘common’ diseases.
